# Common Bean: A Legume Model on the Rise for Unraveling Responses and Adaptations to Iron, Zinc, and Phosphate Deficiencies

**DOI:** 10.3389/fpls.2016.00600

**Published:** 2016-05-03

**Authors:** Norma A. Castro-Guerrero, Mariel C. Isidra-Arellano, David G. Mendoza-Cozatl, Oswaldo Valdés-López

**Affiliations:** ^1^Plant Sciences, Christopher S. Bond Life Sciences Center, University of Missouri, ColumbiaMO, USA; ^2^Laboratorio de Genómica Funcional de Leguminosas, FES Iztacala, Universidad Nacional Autónoma de MéxicoCiudad de México, México

**Keywords:** dry beans production, micronutrients, mineral deficiencies, biofortification

## Abstract

Common bean (*Phaseolus vulgaris*) was domesticated ∼8000 years ago in the Americas and today is a staple food worldwide. Besides caloric intake, common bean is also an important source of protein and micronutrients and it is widely appreciated in developing countries for their affordability (compared to animal protein) and its long storage life. As a legume, common bean also has the economic and environmental benefit of associating with nitrogen-fixing bacteria, thus reducing the use of synthetic fertilizers, which is key for sustainable agriculture. Despite significant advances in the plant nutrition field, the mechanisms underlying the adaptation of common bean to low nutrient input remains largely unknown. The recent release of the common bean genome offers, for the first time, the possibility of applying techniques and approaches that have been exclusive to model plants to study the adaptive responses of common bean to challenging environments. In this review, we discuss the hallmarks of common bean domestication and subsequent distribution around the globe. We also discuss recent advances in phosphate, iron, and zinc homeostasis, as these nutrients often limit plant growth, development, and yield. In addition, iron and zinc are major targets of crop biofortification to improve human nutrition. Developing common bean varieties able to thrive under nutrient limiting conditions will have a major impact on human nutrition, particularly in countries where dry beans are the main source of carbohydrates, protein and minerals.

## Introduction

**FIGURE 1:**
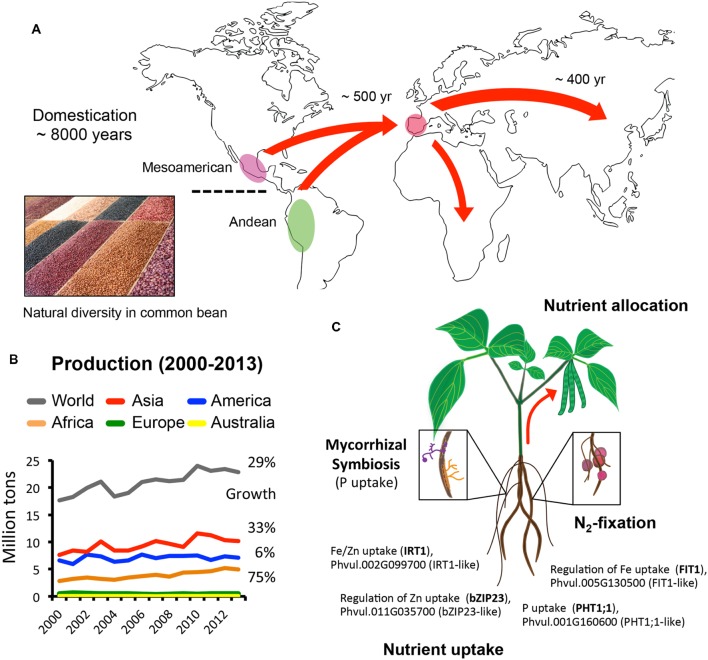
**Domestication, distribution, and production of common bean worldwide.** **(A)** Geographic distribution of common bean following domestication. Insert photo show the natural diversity in common bean in terms of shape, size, and color (Photo by Carlos Adampol Galindo). **(B)** Common bean production (million tons) worldwide in the last decade shows that Africa had the fastest growth. Graph was created using data available at http://faostat3.fao.org. **(C)** Common bean association with symbiotic mycorrhizae and N_2_-fixating bacteria facilitates P uptake and N assimilation. Bottom: for comparison, *Phaseolus vulgaris* proteins with the highest identity, at the amino acid level, to known regulators and transporters of P, Fe, and Zn uptake in *Arabidopsis* are shown (see text and **Table 1** for additional details).

Common bean (*Phaseolus vulgaris*) and soybean (*Glycine max*) are two of the most important sources of protein for humans and livestock. Both legumes have fascinating domestication and diversification histories and today, both are critical pillars of the worldwide economy (**Figure 1**). At the same time, common bean and soybean have different properties that make them attractive to different markets and consumers. For instance, in the USA, Brazil, Argentina, and China, soybean is processed for its protein and oil into a variety of products including food for humans and livestock, cooking oil, and even biofuels. In contrast, common bean is a staple food that does not require any industrial processing; it is the most important protein source in the developing world and, besides the caloric intake, common bean also provides minerals, fiber, thiamine, folate, and phytochemicals with analgesic and neuroprotective properties (Blair et al., 2013; Jha et al., 2015; USDA Nutrient Database release^1^ 19). In 2010, the soybean genome was released allowing the use of sophisticated and elegant molecular tools to study processes that are unique to legumes, such as symbiotic interactions. In 2014, the genome of common bean became available, offering a unique opportunity to compare and understand, at the molecular level, the similarities and differences that make these species so unique and important for human nutrition around the globe. In this mini-review, we briefly recapitulate the history of domestication and distribution of common bean around the world. We also discuss recent advances in phosphate, iron, and zinc metabolism, which are nutrients known to limit plant growth, development and yield. Developing common bean varieties more resilient to nutritional deficiencies will have a major impact on human nutrition, particularly in countries where dry beans are an affordable source of carbohydrates, protein and minerals.

### Economic Importance of Legumes

Crops are susceptible to biotic and abiotic stresses and depending on the severity of the stress and the plant’s ability to tolerate them, yield could be severely affected. In 2012, the production of soybean and corn in the US was reduced by 10 and 28%, respectively, due to a severe drought^2^. On the other hand, pests and nutritional deficiencies have been tackled by an intensive use of fertilizers and pesticides. While this is a short-term solution, the real task for farmers and scientists is to provide food of high nutritional quality compatible with sustainable agricultural practices (Foley et al., 2011). Legumes such as soybean and common bean have the ability to establish symbiotic associations with nitrogen-fixing bacteria (Rhizobia) and with arbuscular mycorrhizal fungi (AM) to facilitate the acquisition of nutrients such as nitrogen and phosphorous (**Figure 1**; Vance, 2001; Venkateshwaran et al., 2013). These symbiotic relationships are economically important because they reduce dramatically the use of synthetic fertilizers and the release of their byproduct N_2_O, a major greenhouse gas. It has been estimated that legumes fix 60 million metric tons of nitrogen worldwide (Smil, 1999) and, replacing this biofixed nitrogen with synthetic fertilizers would cost around $40 US billion (10^9^) dollars. Therefore, understanding and improving these symbiotic associations could have major economic, environmental, and nutritional benefits.

### Genetic Diversity in Common Bean and Genome Hallmarks

Common bean was domesticated ∼8000 years ago in central Mexico and South America (**Figure 1A**). These were individual events and resulted in two major genetic pools, the *Mesoamerican*, covering from northern Mexico to Colombia, and the *Andean*, extending from Peru to Argentina (Gepts, 1998; Rodriguez et al., 2015). Dried beans from both genetic pools were brought to the Iberian peninsula from the *Americas* about 500 years ago and distributed to the rest of Europe, Africa, and Asia shortly after (**Figure 1A**; Santalla et al., 2002, 2010; Angioi et al., 2010). Phaseolin, which accounts for 90% of the seed protein, together with additional molecular and microsatellite markers, have been key to establish the relationship between cultivars, their original genetic pool, and the reduction in the molecular diversity specific to different regions (Gepts, 1998; Broughton et al., 2003; Rodriguez et al., 2015). For example, genetic analysis of European cultivars has showed a higher ratio of *Andean/Mesoamerican* diversity across the continent (Angioi et al., 2010) while in China is the Mesoamerican varieties the most represented. In Africa, there is a mixed abundance of both pools depending on the region (Blair et al., 2010). This vast geographical distribution around the world and the wealth of traits selected over centuries is the result of farming under different environments and the specific needs and preferences of different communities.

Interestingly, the production of dried beans over the last 10 years has increased 29%, for a total of 22 million tons in^3^ 2013. Notably, among all continents, Africa was the one who showed the highest growth in production (75%), compared to Asia (33%) or the Americas (6%; **Figure 1B**). This data trend emphasizes the importance of developing better, more resilient, high-yielding varieties of common bean to mitigate the threat of food insecurity. Breeding programs have been very successful at improving varieties in a location-specific manner and the recent release of a second common bean genome (Mesoamerican BAT93) will likely provide additional information about traits retained in specific geographical regions and their role in adaptation to different environments (Vlasova et al., 2016).

Common bean has a lower genetic complexity relative to soybean. It is known that both species shared a whole-genome duplication (WGD) event about 19 million years ago (Lavin et al., 2005; Schmutz et al., 2010). However, soybean went through a second WGD event 10 million years ago, making soybean a partial diploidized tetraploid. This extra WGD is evident by the number of loci present across the genome and within specific gene families (**Table 1**), thus complicating genetic analyses and mutant identification due to functional redundancies. Synteny analysis, however, showed that most of the common bean genome has counterparts in the soybean genome (Schmutz et al., 2014), opening the possibility to investigate processes in common bean and later pursue cross-species comparisons between these two legumes. Moreover, the comparison of the Andean and the recently released Mesoamerican genome has initially revealed interesting differences. For example, the size and the number of genes in the Mesoamerican genome is 7% smaller (549.6 Mb vs 587 Mb) than the Andean genome. BAT93 has only 30,491 predicted loci, while G19833 has 31,638 (Vlasova et al., 2016). The lower complexity of common bean compared to soybean and the novel genetic information available for the Andean and Mesoamerican pools make common bean an enticing model to characterize molecular processes such as symbiosis and adaptation to nutritional deficiencies.

**Table 1:** Comparison between number of genes and selected functional categories between *Arabidopsis thaliana* (*Arabidopsis*), *Glycine max* (soybean), and *Phaseolus vulgaris* (common bean).

Gene number, family, or annotation	*Arabidopsis*	Soybean	Common bean
Total number of genes (loci)^a^	27,416	56,044	27,197
Amino acid transporter^a^	61	189	96
Oligopeptide transporter^b^	9	24	14
Sulfate transporter (SULTR1-like)^c^	13	44	17
Phosphate transporter (PHT1-like)^c^	19	21	9
Sucrose transporter (SUC2-like)^c^	9	16	9
Nitrate transporter (NRT1-like)^c^	53	86	56
Ammonium transporter (AMT1-like)^c^	6	16	11
Metal transporter^b^	68	161	78
ZIP family, ZIP-like^a^	16	29	20
NRAMP family, NRAMP-like^a^	7	16	7
Iron ion binding (GO:0005506)^a^	346	693	398
Zinc ion binding (GO:0008270)^a^	979	1994	1043
Transcription factors	2492^d^	6667^e^	3726^e^
Receptor^b^	422	754	402
Photoreceptor (blue and red/far-red light)^b^	8	11	7


*^a,b,c^Numbers reflect one gene per locus, not the sum of all possible splice variants. When several variants were available, only the first one (x.1) was considered; GO terms were used only where specified; ^a^Data from Phytozome 10.3; ^b^Based on the current annotation for each genome: TAIR10, *Glycine max* Wm82.a2.v1 or *Phaseolus vulgaris* v1.0 (available through Phytozome 10.3); ^c^Numbers generated using BLASTP in Phytozome and the following gene models: SULTR1; 3 (At1g22150), PHT1; 8 (At1g20860), SUC2 (At1g22710), NRT1 (At1g12110), AMT1; 2 (At1g64780); ^d^Data obtained from Pruneda-Paz et al. (2014); ^e^Data obtained from O’Rourke et al. (2014).*

### Common Bean Responses to Phosphorous, Iron, and Zinc Deficiencies

The symbiotic association of common bean with rhizobia gives an advantage to acquire atmospheric nitrogen, leaving phosphorous (P) as the major limiting macronutrient for plant growth (**Figure 1C**). Most of the components of the phosphorus homeostasis network have been identified in different plants, including common bean (Valdes-Lopez et al., 2008; Lopez-Arredondo et al., 2014). P deficiency promotes changes in root architecture, root exploration capacity and the symbiotic associations with the soil microbiome (Lopez-Arredondo et al., 2014). During P deprivation, common bean is able to use internal phosphate sources like phospholipids, nucleic acids, and ATP (Valdes-Lopez et al., 2008; Lopez-Arredondo et al., 2014). In addition, large-scale metabolomic analyses have shown that during P deficiency there is an accumulation and exudation of organic acids and amino acids into the rhizosphere to solubilize P and increase its uptake (Tawaraya et al., 2014). Phosphate is taken up from the soil by plasma membrane high-affinity phosphate transporters (Pht), which are induced under P deficiency (Valdes-Lopez et al., 2008; Lopez-Arredondo et al., 2014) and remobilized from the plasma membrane when P levels are restored (Bayle et al., 2011). Transcriptomic and metabolite profiling have identified more than 100 genes regulated by P availability in roots and nodules from common bean (Hernandez et al., 2009) and transcription factors such as PHR1 or TIFY, the microRNA PvmiR399 and photosynthates have been identified as part of the P deprivation signaling pathway (Pant et al., 2008; Valdes-Lopez et al., 2008; Liu et al., 2010; Aparicio-Fabre et al., 2013). Shoot-to-root communication is also critical to sense the P status at the whole plant level and it has been suggested that sugars might act as systemic signal molecules that relay information to roots about the plant P status (Liu et al., 2010). Legume–Rhizobium interactions are also important in the context of P homeostasis. In nodules, the P concentration is higher than the rest of the plant, even under P stress, suggesting that nodules play a major role regulating the C/N flux between the plant and the bacteria (Hernandez et al., 2009). In this regard, the transcription factors NF-YC1 and AP2, and the microRNA miR172 were found to regulate the Rhizobia symbiosis, paving the road for improving N-fixation in common bean (Nova-Franco et al., 2015), while optimizing P uptake simultaneously. Other associations such as endomycorrhizal symbiosis (**Figure 1C**) seem to facilitate P assimilation, but the molecular mechanisms regulating this interaction are still largely unknown in common bean.

Micronutrient deficiencies, particularly Fe and Zn, affect over 30% of the world’s population^4^. These essential minerals are required in several metabolic processes. For example, in humans, iron is needed to synthesize hemoglobin, hormones, and connective tissue; while zinc is required as an enzyme co-factor, for immunity, protein synthesis, DNA synthesis, and cell division. Women, particularly pregnant woman, and children in developing countries are particularly at risk due to poor micronutrient intake. Common bean is a staple food in developing countries and also a good source for these minerals having more iron (around 55 μg/g) and zinc (around 35 μg/g) than cereals (Beebe et al., 2000a). Although there is some correlation between Fe and Zn across common bean cultivars (Hoppler et al., 2014), the Andean pool seem to have higher concentration of Fe (Beebe et al., 2000b) while the Mesoamerican beans have higher zinc content (Islam et al., 2002). These essential nutrients are taken up from the rhizosphere by transporters that have already been identified and characterized (**Table 1**). However, even in the model plant *Arabidopsis*, the transporters responsible for the mobilization and loading of nutrients into seeds remain largely unknown. Fe and Zn deficiency in plants is caused by low availability or solubility of these elements in the soil. In Strategy I plants, like common bean, release of protons by the H+ATPases, specially AHA2, induces an acidification of the rhizosphere, which in turn promotes the solubilization of Fe and Zn thus facilitating their uptake (Guerinot and Yi, 1994; Marschner and Romheld, 1994; Sinclair and Kramer, 2012). Both, Fe and Zn are taken up by IRT-like transporters (Hindt and Guerinot, 2012; Sinclair and Kramer, 2012) and then mobilized to the shoots via the xylem. Source-to-sink transport on the other hand, including nutrient delivery to developing tissues including seeds, occurs exclusively via the phloem (Mendoza-Cozatl et al., 2011; Khan et al., 2014). Surprisingly, little is known about how Fe and Zn are sensed in plants. But downstream transcriptional regulators for both elements have been identified in *Arabidopsis*. Up regulation of the Fe uptake machinery is under the control of two major transcriptional networks, the FIT (At2g28160) network and the PYE (At3g47640) network, while Zn uptake during Zn deprivation requires two bZIP transcription factors, bZIP19 and bZIP23 (Hindt and Guerinot, 2012; Sinclair and Kramer, 2012). An additional protein localized in the phloem, OPT3, has been identified as a component of the shoot-to-root signaling network relaying the information of the Fe status in leaves to roots (Mendoza-Cozatl et al., 2014; Zhai et al., 2014). Interestingly, since Fe and Zn share the same transporters to enter the root, mutants with impaired systemic signaling (*opt3* mutants) over accumulate Fe and Zn in roots and leaves (Mendoza-Cozatl et al., 2014; Zhai et al., 2014). Moreover, the common bean genome contains putative homologs for all the components of these networks (Phvul.005G130500/FIT1-like; Phvul.002G099700/IRT1-like; Phvul.003G086500/OPT3-like; Phvul.011G035700/bZIP23-like), suggesting that current advances in trace metal homeostasis in *Arabidopsis* could have a direct translational impact in common bean and could lead to an increased allocation and retention of Fe and Zn in the edible plant tissues.

Biofortification in common bean needs an adequate partitioning of Fe and Zn between plant tissues. Breeding approaches have already achieved Fe concentrations in beans of up to 94 μg/g (Petry et al., 2015); however, the underlying molecular mechanisms behind this feat are unknown. Natural variation plays a major role in identifying and breeding desirable traits into varieties amenable to specific climates. In seeds, Fe is stored in ferritin complexes (Theil, 2003), thus ferritin has been the focus for Fe biofortification in other plant species. But only a portion of the iron in ferritin is bioavailable; therefore, alternative strategies to accumulate Fe in bioavailable forms are currently being investigated (Hoppler et al., 2014). Among them are small Fe and Zn binding molecules such as nicotianamine and organic acids found in vacuoles like malate and citrate. Another target has been to reduce anti-nutrients like phytic acid and polyphenols, which bind to Fe and Zn thus inhibiting their absorption (Clemens, 2014; Petry et al., 2015). An additional challenge is to have an increase in Fe and Zn levels without compromising the content of other nutrients or increasing the uptake of non-essential toxic elements such as Cd and Ni. These elements can occur naturally in trace amounts but these elements use the Fe/Zn uptake mechanism (IRT1-mediated) to enter the roots (Mendoza-Cozatl et al., 2011; Khan et al., 2014).

### Opportunities to Maximize Nutrient Acquisition in Common Bean

*Arabidopsis thaliana* was originally selected as a model plant based on its relatively small genome. *Arabidopsis* has been, over the last 20 years, the go-to model to characterize genes and assign putative functions to more than 35,000 hypothetical proteins encoded by 27,000+ loci. *Arabidopsis* has been key to understand complex molecular processes in plants, and the advances achieved so far have been extraordinary in terms of mechanistic details. Surprisingly, the number of genes (loci) in common bean is not significantly different than *Arabidopsis* (**Table 1**) and the number of members within gene families – from specific transporters, to signaling proteins and transcription factors – is pretty close between these two species. These similarities suggest that it is feasible to use the accumulated knowledge in *Arabidopsis* as a *bona fide* reference to explore processes of specific interest in common bean (e.g., nutrient uptake and allocation) but also to start unraveling the molecular basis of processes more unique to legumes (e.g., nodulation). In addition, the *Arabidopsis* genome can also be used as a “contrasting reference” to explore additional symbiotic interactions, particularly with AM fungi. Comparative phylogenomic analyses have found that *Arabidopsis*, together with a handful of other species, has lost some of the genes required to establish successful interactions with AM. The molecular basis for this loss is unclear but it has been suggested that the emergence of new traits allowing a more efficient nutrient uptake decreased the selection pressure to maintain genes critical for symbiosis with AM (Delaux et al., 2014).

An important yet less fortunate difference between *Arabidopsis* and common bean is the current difficulty to stably transform common bean and modify gene expression (either over expression or silencing). Therefore, transformation in common bean is mostly limited to hairy roots; that is, genes can be successfully over expressed or silenced but only in root tissues and within one generation. Nevertheless, hairy root transformation is efficient enough to enable functional genetic approaches (Estrada-Navarrete et al., 2007), including the use of artificial microRNAs to silence simultaneously several genes within a gene family.

Another key resource available to help identifying genes and networks expressed in a tissue-specific manner is the *P. vulgaris* Gene Expression Atlas database^5^ (O’Rourke et al., 2014). This database contains expression profiles, obtained by RNA-seq using the variety *Negro Jamapa*, from seven tissues including roots, nodules, stems, flowers, leaves, pods, and seeds throughout development. These bioinformatic resources are critical to integrate transcriptional programs in complex organisms subject to specific developmental programs, like plants. Since gene annotation and assignment of protein function is an ongoing process, tissue-specific datasets are extremely useful to narrow down candidate genes for further studies. For instance, once a transporter gene is suspected to mediate the mobilization of nutrients into seeds, based on homology to other nutrient transporters and its unique expression at seed-filling stages, this protein can be cloned and expressed in heterologous systems (e.g., yeast or oocytes) for a detailed biochemical characterization including substrate specificity (Mendoza-Cozatl et al., 2014; Zhai et al., 2014).

Perhaps, one of the most breakthrough technologies brought to the plant field in the last decade is the tag-based immunoprecipitation of translating ribosomes (or TRAP). This technique recovers mRNA associated to ribosomes to assess changes in gene translation (translatome analyses). Due to transgenic nature of this approach (i.e., the tag used for immunoprecipitation is fused to the ribosome by standard cloning), tissue-specific promoters can be used to drive the expression of the tagged ribosome in specific tissues, making translatome analysis the method of choice to identify genes being translated in specific cells or tissues. The TRAP technique has recently been adapted to legumes and will likely advance our understanding of molecular processes at cell- and tissue-specific resolution (Castro-Guerrero et al., 2016). The availability of the common bean genome is also facilitating other *–omic* techniques including epigenomics (Kim et al., 2015), small RNA and degradome sequencing (Formey et al., 2015) and will likely enable proteomic studies in the near future.

### Perspectives and Conclusion

The recent release of the Andean and Mesoamerican common bean genomes is enabling a new wave of cutting-edge research, including epigenomics and translatome analyses, in a crop that has fed billions of people for more than 5000 years. The tools developed in other crops to harness the power of natural diversity will play a major role at identifying traits needed to overcome biotic and abiotic stresses. These are exciting times for a field that has the potential to reduce the threat of food insecurity by engineering crops tolerant to biotic and abiotic stresses, increasing yields and enhancing the nutritional quality of dried beans.

## Author Contributions

DM-C, and OV-L conceived the idea. NC-G, MI-A, DM-C, and OV-L performed the bibliographic survey. NC-G, MI-A, DM-C, and OV-L wrote the paper.

## Conflict of Interest Statement

The authors declare that the research was conducted in the absence of any commercial or financial relationships that could be construed as a potential conflict of interest.

## Footnotes

1 https://ndb.nal.usda.gov

2 http://www.ers.usda.gov/topics/in-the-news/us-drought-2012-farm-and-food-impacts/.aspx

3 http://faostat3.fao.org

4 http://www.who.int/nutrition/topics/ida/en/

5 plantgrn.noble.org/PvGEA/
